# Usefulness of the radiological planning for hearing preservation surgery in vestibular schwannoma

**DOI:** 10.1007/s00276-016-1668-z

**Published:** 2016-03-22

**Authors:** Kamil Krystkiewicz, Tymon Skadorwa, Paweł Szaro, Bogdan Ciszek

**Affiliations:** 1 Department of Descriptive and Clinical Anatomy, Medical University of Warsaw, Chałubińskiego 5, Warsaw, 02-004 Poland; 2 Department of Pediatric Neurosurgery, Bogdanowicz Memorial Hospital for Children, Warsaw, Poland; 3 1st Department of Clinical Radiology, Medical University of Warsaw, Warsaw, Poland

**Keywords:** Vestibular schwannoma, Endolymphatic duct, Endolymphatic sac, Hearing preservation surgery, Retrosigmoid approach

## Abstract

**Purpose:**

During vestibular schwannoma surgery there is a risk of endolymphatic duct and sac injury, which may cause a loss or a deterioration of hearing. The goal of the study was to evaluate the empirical utility of presurgical planning using CT with the bone window for the hearing preservation surgery.

**Methods:**

The study was performed on 14 human temporal bones. CT scans with the bone window were obtained in the standard position. Safe drilling line was evaluated and after that drilling distances were analysed: the lateral drilling distance, total length of internal acoustic meatus, drilled length of internal acoustic meatus. After this, a surgical exposure was performed, using size of a drill tip as measuring scale. The dura was excised and endolymphatic duct was injected with a latex. Revision of the internal acoustic meatus was performed with the use of a microscope.

**Results:**

Mean results of safe drilling coefficients were: lateral drilling distance: 10 ± 2 mm, total length of internal acoustic meatus: 9 ± 2 mm, drilled length of internal acoustic meatus: 7 ± 2 mm. In all cases, no endolymphatic duct injury was observed.

**Conclusions:**

Preoperative radiological planning using the safe drilling coefficients is of value for the hearing preservation surgery in vestibular schwannoma. The size of the drilling tip may be used as an intraoperative measuring scale during this procedure. However, CT with a bone window is a necessary tool for the purposes of this procedure.

## Introduction

Hearing preservation is one of the goals during the surgery of vestibular schwannoma (VS). It is achievable through a suboccipital retrosigmoid approach, when the preoperative hearing was determined as usable. This aim is fulfilled even in 51 % [9] and depends on multiple factors. An immediate hearing loss during a surgery is explained as a result of: (1) a cochlear nerve damage during tumour separation from a nerve; (2) a vestibule or cochlea injury during drilling; (3) disrupted vascular supply to the inner ear. However, a hearing functionality in the short term after the surgery may worsen in the postoperative observation. A deterioration of hearing was observed by many authors [1, 2, 6, 9]. The frequency of this fact has a wide range from 0 to 56 % [1, 2, 6, 9]. There exist a few explanations of this phenomenon. One of them is a damage of the endolymphatic duct (ED) or sac (ES) during removal of the posterior wall of the internal acoustic meatus (IAM) [1, 2, 6, 9]. Some authors observed that the obstruction of the endolymph flow through ED causes slow, but progressive hearing deterioration and symptoms similar to Meniere’s disease. These observations empower the hypothesis that damaging the ED or the ES could be a significant cause of progressive hearing deterioration after the VS resection through the suboccipital retrosigmoid approach. Preoperative planning in our opinion might help in determining the range of safe IAM exposure. Therefore the aim of our study was to evaluate the accuracy of a radiological analysis for a planned exposure of the IAM when the ED and ES preservation is targeted.

## Methods

In the study, 14 specimens of human temporal bones (ten left, four right) covered with dura and with natural contents of the bony canals were used. None of the specimens had a visible pathology. All examinations were performed on the Toshiba Aquilion 64 CT Scanner. Samples were placed in a neutral position similar to the standard examination of that area for clinical purposes. Coordinates were established on the ‘temporal bone’ preset, with the slide thickness of 0.5 mm. After this, the radiological analysis was performed with the use of the eFilm™ Lite™ software. The preoperative planning needed measurements as described below in two steps. Posterior wall of a temporal bone was analysed regarding ED topography. The medialmost point of ED was considered as a lateral limitation of area of safe drilling. Next, from this point the line was drawn rearward to meet a point of standard dural incision—2 cm medial to the edge of sulcus of sigmoid sinus (SSS) [4]. Thus, a “safe drilling line” (SDL) was designed. This line provides boundaries of the safe drilling area of the temporal bone. It is believed that the risk of damage of ED is minimal in the area located medially from this line. Safe drilling area in CT examination was also inspected for anatomical variations and verified during the anatomical procedure.

**Fig. 1:**
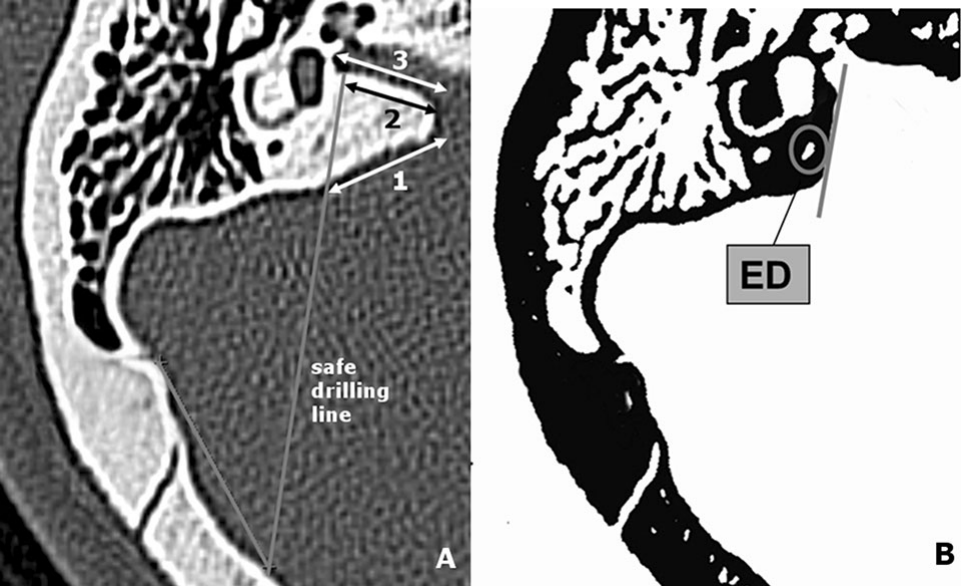
**a** The measurements technique presented on the right temporal bone. (1) Lateral limitation of the drilling of posterior wall of petrous bone (LL), (2) Drilled length of internal acoustic meatus (D-IAM), (3) Total length of internal acoustic meatus (T-IAM). **b** The status of endolymphatic duct after procedure. *ED* endolymphatic duct

Thanks to SDL we designed some parameters to provide a valuable information for the neurosurgeon about the safe distance of drilling. First of them—the lateral limitation of the posterior wall of petrous bone (LL) was a distance from the posterior margin of internal acoustic porus (PMP) to the SDL (Fig. 1). The second parameter was a distance from the most external point of PMP to the SDL measured along the posterior wall of IAM—the drilled IAM (D–IAM). The third was a distance from the same point to the fundus of the meatus—the total length of IAM (T–IAM). Basing on the last two distances the percentage ratio of IAM exposure was calculated with the formula: (D-IAM/T-IAM) × 100 % (Coeff.)

**Fig. 2:**
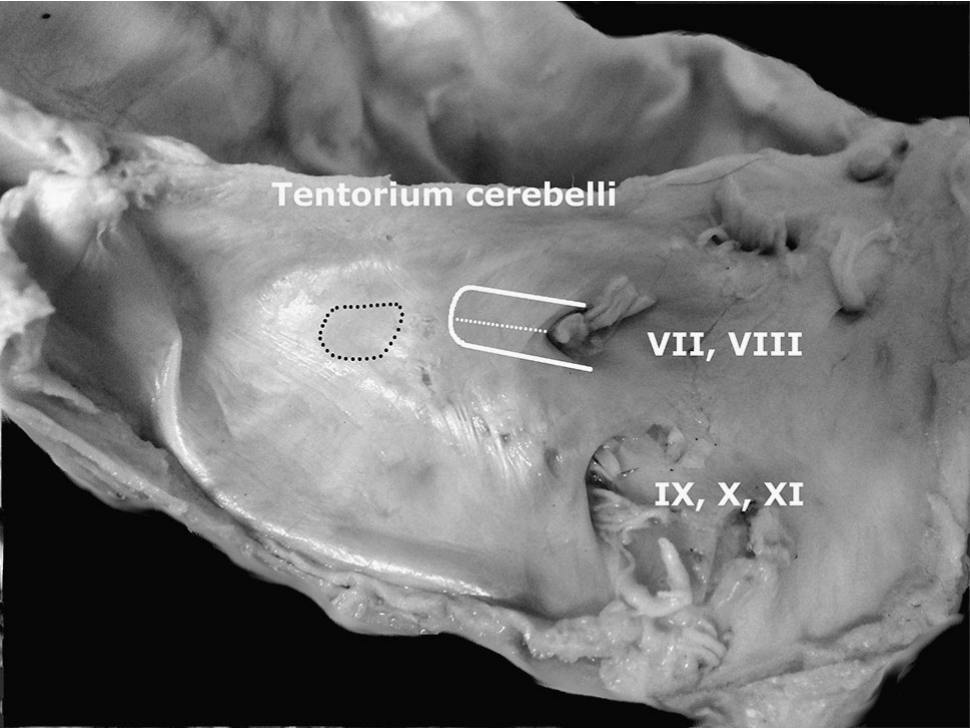
The anatomy of the posterior wall of left temporal bone. *Black doted area*, the location of the endolymphatic sac covered with dura. *White lined area*, dural flap and extent of drilling. *White dotted line*, the lateral limitation of the drilling of posterior wall of petrous bone (LL)

After the planning, a bone specimen was placed in a special holder in position similar to the one observed in the surgical field. The angle of approach was set to 25°, which was based on previous measurements between SDL and the surface of posterior wall of petrous bone. Using an intraoperative microscope, a dura mater was cut in the standard fashion on the length of LL (Fig. 2). ES was identified and ED was injected with coloured latex through the 22G cannula. Using the drill tip (5 mm) as an intraoperative scale, the bone was then drilled on the length previously measured in CT scans. After this procedure, the IAM and its walls were revised for the eventual ED injury. Finally, the measurements of LL, D-IAM and T-IAM were performed on the specimen and compared with the radiological part of the study.

## Results

The drilling was intentionally conducted according to previous radiological distances to assess their accuracy. All the measured distances are presented in Table 1. From the measured parameters, a LL seems to be the most valuable for a neurosurgeon. Despite a minimal difference in its value in radiological planning and anatomical inspection, this distance provided a 74 % opening of the IAM in both parts of the study. The remaining parameters did not present statistically significant differences between radiological and anatomical parts (*p* > 0.05).

**Table 1:** Parameters measured in radiological and anatomical part of the study

	Radiological		Anatomical	
	Avg ± SD	Min–max	Avg ± SD	Min–max
LL (mm)	11 ± 2	8–13	10 ± 1	8–13
D-IAM (mm)	7 ± 2	6–8	6 ± 1	4–9
T-IAM (mm)	9 ± 2	2–7	8 ± 2	2–7
Coeff. (%)	74 ± 10	58–86	74 ± 14	50–88

Parameters measured in radiological and anatomical part of the study)

*LL* lateral limitation of the posterior wall of petrous bone, *D-IAM* drilled IAM, *T-IAM* total length of IAM, *Coeff.* percentage ratio of IAM exposure (see in the text)

**Fig. 3:**
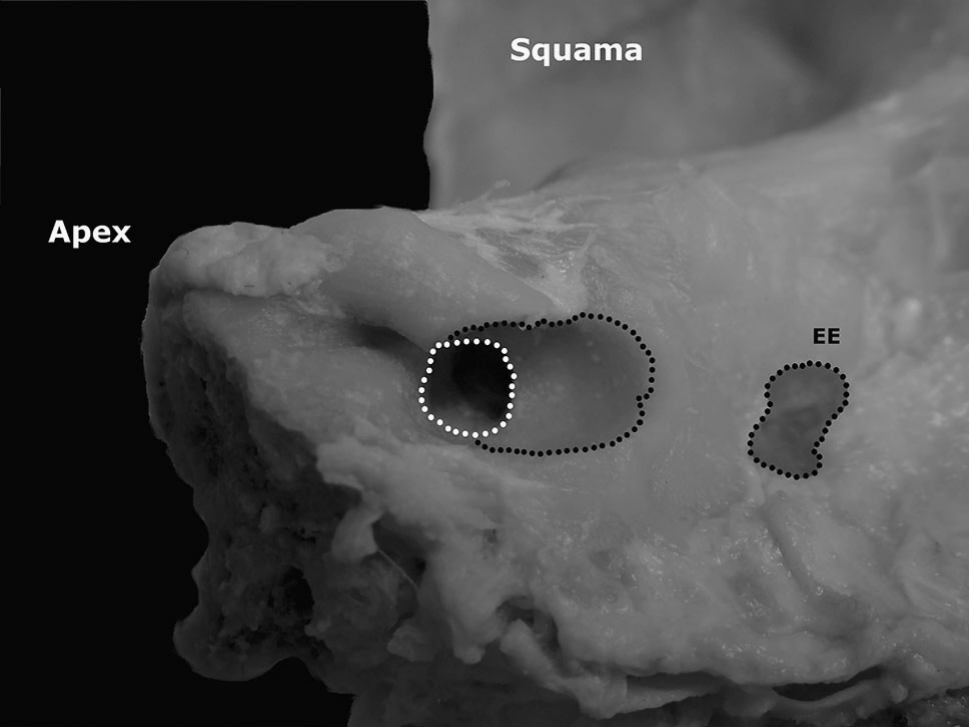
The specimen of right temporal bone after the procedure. No endolymphatic duct can be observed in the area of drilling. *EE* endolymphatic excavation, where the endolymphatic sac could be found after the dissection of dura

**Fig. 4:**
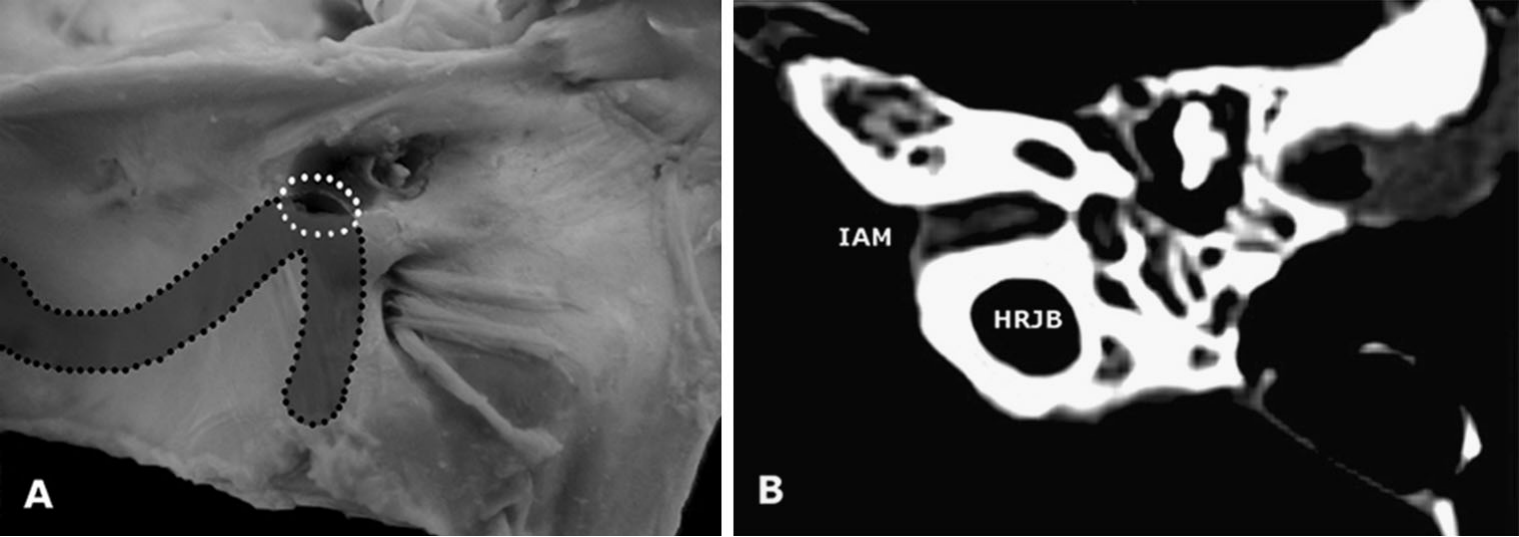
**a** The case of high riding jugular bulb in the specimen of left temporal bone. *White dotted area*, injured jugular bulb visible in area of drilling. *Highlighted* and *black doted area*, the projection of the course of the sigmoid sinus. **b** A preprocedural CT examination of the same specimen. It is visible, that jugular bulb is located posteriorly to the internal acoustic meatus. *HRJB* high riding jugular bulb, *IAM* internal acoustic meatus

A microscopic inspection of a drilled area revealed no ED injury in all of the cases (Fig. 3). Instead, we found mastoid air cells violation in two cases, which was a most common intraoperative complication. In one case there was visible the presence of high riding jugular bulb in the safe drilling area in the CT examination. In that particular case procedure was planned and provided to assess the maximum exposure of IAM without damaging the ED despite the jugular bulb in the trajectory of drilling (Fig. 4). During the anatomical part of study the jugular bulb was visible and intentionally injured in case to confirm the usefulness of CT examinations in detecting such anatomical variations in the safe drilling area.

## Discussion

The relation of ES and ED to adjacent vascular and bony elements has been discussed in the literature [5, 11]. The anatomical position of ES and ED promotes their possible intraoperative damage. Resulting symptoms are similar to Meniere’s disease. Park et al. [7] analysed the HRCT scans of 167 patients with Meniere’s disease. A control group comprised studies of healthy individuals without the inner ear pathology. The authors found significant that the high riding jugular bulb was more common in the Meniere’s disease group than in the control group (21 % vs. 13.3 %). What is more, ED was the closest structure of the inner ear to the jugular bulb (7.5 %). Other studies determine the relation between ED or ES obstruction and a slow progressive hearing deterioration. Rizvi and Gibbin [8] analysed five temporal bones after a transverse fracture. In four of them the fracture line traversed the ED in CT scans. One of the patients, with the complete obstruction of the ED manifested the symptoms of Meniere’s disease. Jeffrey et al. [3] analysed 31 patients with ES tumour. 29 patients were symptomatic and presented the symptoms of Meniere’s disease, such as sensorineural hearing loss (84 %), tinnitus and vertigo. These studies reveal that Meniere’s disease is a multifactor disorder and cannot be explained with a unique anatomical abnormality. Anatomical variants of a temporal bone and surrounding vessels may, however, play a significant role in a pathophysiology of a hearing loss.

Clinical observations prove a hearing preservation to be one of the most challenging targets to achieve during the VS surgery. The outcome, however, may not be satisfactory in long-term observation. In some of published case series, the deterioration of hearing was reported regardless the size of a tumour and despite the preservation of usable hearing during surgery. Betchen et al. [2] analysed retrospectively 142 patients after the VS resection. Thirty-five patients with immediate postoperative audiogram tests were examined again with a mean time of 7 years after the surgery. The hearing deterioration was observed in 14.3 % of cases. Similar results were presented by Nakamizo et al. [6]. From 57 patients with the serviceable preoperative hearing, 28 retained the hearing after surgery. From this group, 24 patients were observed for an average period of 68.8 months. In the follow-up period 17 % of operated patients presented a deterioration of hearing despite the favorable initial postoperative audiogram. The authors did not find a significant hearing decline in the non-operated ear. Despite numerous studies on this problematic topic, still a precise mechanism of a progressive hearing loss following surgery of VS is not known.

One of the hypotheses puts an emphasis on the significance of preserving the ED during IAM drilling. Sulman et al. [10] dissected 21 human temporal bones for the analysis of ED or ES injury during a suboccipital approach. The material was examined with the use of HRCT. Subsequently, a typical exposure of the IAM was performed with the intraoperative preservation of the ES, the vestibule and the posterior semicircular canal. After the procedure, bones were re-examined with the HRCT for the presence of possible ED injury. Five out of 21 petrous bones (24 %) presented the ED violation, without the damage of vestibule or other inner ear structures. The authors, however, did not provide any quantitative data in their research. We progressed one step further on and used measurements and coefficients based on pre-procedural CT of our specimens in order to evaluate an individual range of the safe IAM exposure. The injection of coloured latex, clearly visible in the operative microscope, enabled a sufficient revision of the IAM in search for a possible ED violation.

A study we performed allows in our opinion to prove the accuracy of radiological preoperative assessment. Although it does not provide arbitrary values for safe IAM drilling, which results from a limited number of specimens, it presents a spectrum of parameters and coefficients that can be used during planning. The evaluation of the safe drilling area seems to be crucial for operative technique, as scheduled distances create conditions for larger and safer exploration of the IAM. However, every approach needs to be set accordingly to individual anatomical configuration. Therefore, the accuracy of radiological measurements proved in direct anatomical reassessment, in our opinion makes CT a mandatory diagnostic tool before every VS surgery.

## Conclusions

The study revealed that the described methodology of the CT analysis is useful in evaluating the drilling area where there is no risk of an ED injury. The accuracy of performed measurements was assessed empirically and in our opinion preoperative planning should be mandatory for the hearing preservation VS surgery. A CT with a bone window should be an obligatory examination, because it provides a crucial information for a safe and uncomplicated VS resection.

## References

[1] Atlas MD, Harvey C, Fagan PA (1992). Hearing preservation in acoustic neuroma surgery: a continuing study. Laryngoscope.

[2] Betchen SA, Walsh J (2005). Post KD (2005) Long-term hearing preservation after surgery for vestibular schwannoma. J Neurosurg.

[3] Jeffrey Kim H, Hagan M, Butman JA (2013). Surgical resection of endolymphatic sac tumors in von Hippel-Lindau disease: findings, results, and indications. Laryngoscope.

[4] Martuza RL, Quiñones-Hinojosa A (2012). Suboccipital Retrosigmoid Surgical Approach for Vestibular Schwannoma (Acoustic Neuroma). Schmidek & Sweet operative neurosurgical techniques: Indications, methods, and results.

[5] Mutlu C, Govsa F, Unlu HH, Senyilmaz Y (1997). The variational anatomy of the external aperture of the human vestibular aqueduct. Surg Radiol Anat.

[6] Nakamizo A, Mori M, Inoue D (2013). Long-term hearing outcome after retrosigmoid removal of vestibular schwannoma. Neurol Med Chir.

[7] Park JJ, Shen A, Keil S, Kuhl C, Westhofen M (2015). Jugular bulb abnormalities in patients with Meniere’s disease using high-resolution computed tomography. Eur Arch Otorhinolaryngol.

[8] Rizvi SS, Gibbin KP (1979). Effect of transverse temporal bone fracture on the fluid compartment of the inner ear. Ann Otol Rhinol Laryngol.

[9] Samii M, Gerganov V, Samii A (2006). Improved preservation of hearing and facial nerve function in vestibular schwannoma surgery via the retrosigmoid approach in a series of 200 patients. J Neurosurg.

[10] Sulman CG, Vecchiotti MA, Semaan MT, Lewin JS, Megerian CA (2004). Endolymphatic duct violation during retrosigmoid dissection of the internal auditory canal: a human temporal bone radiographic study. Laryngoscope.

[11] Zhen J, Liu C, Wang S, Liu S, He J, Wang J, Chen H (2007). The thin sectional anatomy of the temporal bone correlated with multislice spiral CT. Surg Radiol Anat.

